# Children's Hospital, Nottingham

**Published:** 1899-02-11

**Authors:** Geoffrey W. Thompson


Dr. GEOFFREY W. THOMPSON, M.D. (Cantab), writes: I have read the article on "The Children's Hospital at Nottingham" and the correspondence about that institution in your paper, THE HOSPITAL, for January 21st, 1899. In 1893 I was house surgeon at the Children's Hospital, Nottingham, for nine months, during which time I had every opportunity of knowing how the hospital was managed by the Board of Management, how the nursing was arranged by the sisters and lady superintendent, how the nursing was carried out, and how the medical staff performed their various duties. No fault could be found in any of these arrangements, and the hospital results speak for the excellent work done there. I can truly say I never remember a single complaint being raised against the hospital by any parent of the in or out patients. On the other hand, the mothers and fathers of the inmates were always full of thanks to and praise of the institution. I consider the nursing was carried out in a most perfect and methodical way. I never heard any complaint from the visiting staff as to the nursing, and I certainly never have had, while I was house surgeon and house physician at St. Thomas's Hospital, London, or in private practice, more careful, painstaking, and skilful nurses in charge of my patients. While I was at Nottingham Children's Hospital the isolation block was generally full. The nursing staff was comparatively small for the huge amount of work that the hospital got through, but, nevertheless, the lady superintendent always managed to spare special nurses for the isolation cases. I daresay many of these were probationers, but that made no difference, as the sister in charge was frequently in the block and always supervising the work there. The nurses at isolation at that time certainly never hesitated to ring the bell if they required assistance, and the sister came from the main block to help them at once. Each nurse in isolation had, at that time, a bed in a separate room to her patient, a table on which to take her meals, and a basin in which to wash. Besides this, it always appeared to me that the sister was continually relieving the isolation nurses, in order to send them out for fresh air. I cannot believe that any patient in this institution has ever been neglected in any way, and that the so-called hardships have been exaggerated, and that the abuse indicated never existed.

